# MiR-451 as a new tumor marker for gastric cancer

**DOI:** 10.18632/oncotarget.17239

**Published:** 2017-04-19

**Authors:** Yong Shen, Jiao-Mei Gong, Li-Li Zhou, Jia-He Sheng

**Affiliations:** ^1^ Department of Clinical Laboratory, Affiliated Cancer Hospital of Zhengzhou University, Zhengzhou, Henan, China; ^2^ Department of Pathology and Pathophysiology, Medical School of Southeast University, Nanjing, Jiangsu, China; ^3^ Department of Clinical Laboratory, The Second Affiliated Hospital of Zhengzhou University, Zhengzhou, Henan, China; ^4^ Department of Hepatobiliary Surgery, Affiliated Cancer Hospital of Zhengzhou University, Zhengzhou, Henan, China

**Keywords:** MiR-451, gastric cancer, diagnoses, pathology

## Abstract

Gastric cancer is the second most common malignancy in China. However, the prognosis for gastric cancer patients remains poor. The purpose of this study was to investigate whether miR-451 was a potential prognostic biomarker for gastric cancer. Fresh tissues were immediately frozen in liquid nitrogen until use. The plasma was extracted and quantitative real-time polymerase chain reaction was performed to detect miR-451 expression. The Student's t test analysis and multivariate Cox regression analysis were performed to analyze expression of miR-451. The analysis results showed that the expression level of miR-451 was decreased expression in gastric cancer tissue. The down-regulation of miR-451 tended to be positively correlated with tumor stage, lymphatic metastasis and shorter overall survival of patients. MiR-451 may be a potential biomarker and a potential therapeutic target for the diagnoses and prognosis of gastric cancer.

## INTRODUCTION

Gastric cancer is the second most frequently diagnosed cancer in the world, which is also the leading cause of cancer-related mortalities [1]. In the early stage, the clinical manifestation of gastric cancer is not easily detected, which results in metastasis at diagnosis. The clinical outcome of advanced gastric cancer patients is extremely poor [2–3]. Unfortunately, biomarkers with high sensitivity and specificity for gastric cancer are still lacking. Thus, identifying novel biomarkers for early detection and prediction of the prognosis of gastric cancer is important.

MicroRNAs (miRNAs), small noncoding short RNAs of 18-25 nucleotides in length, generally act as negative regulators of gene expression at post-transcriptional level through mRNA degradation or translation repression [4]. These molecules play important roles in many important biological processes, including cell growth, apoptosis, viral infection and cancer development [5–6]. Increasing evidence has shown that miRNAs can act as oncogenes or tumor suppressor genes, which depend on the genes they modulate [6–7]. For example, miR-153 was down-regulated in gastric cancer tissue and the low expression of miR-153 was correlated with poor prognostic features [8]. MiR-374a was over-expressed in human gastric cancer cell line and tissue [9]. However, as far as we know, there was no study concentrating on the expression levels of miR-451 in gastric cancer.

In this study, we investigated the expression of miR-451 in gastric cancer tissue and explored the association of miR-451 expression with the clinical pathological features data and prognosis of gastric cancer patients.

## RESULTS

### The expression of MiR-451 in patient characteristics

The expression of miR-451 in 152 normal patients was 0.0142±0.0093 (Table 1). However, the expression of miR-451 in gastric cancer group was 0.0105±0.0082. There were significant differences between the two groups (*P*=0.0001). As shown in Table 1, there was no significant difference in the distribution of gender (*P* = 0.6103), age (*P* = 0.5388) and tumor size (*P* = 0.1792). The expression of miR-451 in gastric carcinoma is correlated to tumor stage and lymph node metastasis. At the gastric cancer I/II stage, the expression of miR-451 was 0.0122±0.0048. While the expression of miR-451 was 0.0083±0.0061 at gastric cancer III/IV stage. There was significant difference between the tumor stage (*P*=0.0001). The expression of miR-451 was down-regulated in gastric cancer tissues. Compared with patients who suffer from gastric cancer without lymph node metastasis, the expression of miR-451 was decreased in the tumor of patients with lymph node metastasis (0.0128±0.00510 to 0081±0.0063, *P*=0.0001).

**Table 1:** The expression of miRNA-451 in clinical pathological factors

Clinical Characteristics	Number	The Expression of miRNA-451	P
Gender			
Male	228	0.0105±0.0071	0.6103
Female	40	0.0107±0.0075	
Age (years)			
≤50	105	0.0109±0.0052	0.5388
>50	163	0.0104±0.0072	
Tumor stage(invasion depth)			
I,II	123	0.0122±0.0048	0.0001
III,IV	145	0.0083±0.0061	
Diameter (cm)			
≤4	162	0.0105±0.0042	0.1792
>4	106	0.0098±0.0041	
Lymph node metastasis			
No	120	0.0128±0.0051	0.0001
Yes	148	0.0081±0.0063	
Controls			
Normal	152	0.0142±0.0093	0.0001*

* The *P* value is the average expression level of miRNA-451 between gastric cancer group and control group.

### The expression of MiR-451 in prognosis of patients with gastric cancer

The expression of miR-451 for survival curves was estimated by the Kaplan-Meier method. Compared with the gastric cancer patients who received increased expression of miR-451, the patients in whom miR-451 was down-regulated had shorter survival time (median survival time 44 months vs. 59 months, *P*=0.0001). The low expression of miR-451 was correlated with poor prognostic features.

Multivariate Cox regression analysis revealed that the expression of miR-451 differentiation (hazard ratio (HR)=2.215, 95% confidence interval (CI)=1.698-4.241, *P*=0.0001), tumor stage (HR=1.796, 95%CI=1.178-2.462, *P*=0.0016), and lymph node metastasis (HR =1.802, 95%CI=1.324-2.187, *P*=0.0024) were independent risk factors for gastric cancer (Table 2).

**Table 2:** Multivariate analysis for prognosis of gastric cancer patients

Clinical Characteristics	Group	Survival time		
		HR	95%CI	*P*
Expression of miRNA-451	High/Low	2.215	1.698∼4.241	0.0001
Gender	Male/Female	1.116	0.901∼1.502	0.4582
Age	≤50/>50	1.058	0.911∼1.184	0.6247
Clinical stage	I,II/III,IV	1.796	1.178∼2.462	0.0016
Diameter	≤4cm/>4cm	1.863	1.241∼3.027	0.3475
Lymph node metastasis	No/Yes	1.802	1.324∼2.187	0.0024

Abbreviation: HR, Hazard Ratio; CI, Confidence Interval; CI, Confidence Interval.

## DISCUSSION

Gastric cancer is a heterogeneous, multi-factorial disease with high incidence in China [10]. Although great improvement in treatment has lowered the mortality rate of gastric cancer over recent decades [11]. Unfortunately, the prognosis for advanced gastric cancer patients remains poor and the molecular mechanism of gastric cancer was still poorly understood [12–13]. Therefore, miRNAs had gained much attention due to its important role in carcinogenesis which may bring new options for diagnosis and treatment of gastric cancer [14–15]. In addition, the accurate function of miRNAs *in vivo* still needed further investigation [16].

In this study, we found that decreased expression of miR-451 in gastric cancer was associated with the tumor stage and lymph node metastasis. The result suggested that miR-451 may be a potential biomarker for diagnoses and prognoses of gastric cancer. In addition, our study showed that the expression difference of miR-451 and lymph node metastasis were respectively independent prognostic factors in gastric cancer patients by multivariate analysis. This retrospective analysis suggested that miR-451 could be a prognostic indicator to monitor the recurrence of gastric cancer. According to the authors' knowledge, this is the first study regarding the function of miR-451 in gastric cancer.

Several studies showed that, miRNA-451 played a very important role in the development of cancer [17–18]. The decreased expression of miR-451 was associated with shorter survival in migrating glioblastoma patients by Godlewski et al [18]. The result was similar to ours. However, another study showed that the increased expression of miR-451 could inhibit cell growth and promote cell apoptosis [19]. MiR-451 could also function as a tumor suppressor in human non-small cell lung cancer (NSLC) [20]. In human colorectal carcinoma and hepatocellular carcinoma, miR-451 could inhibit cell proliferation through the down-regulation of PI3k/Akt pathway and direct suppression [21]. The macrophage migration inhibitory factor (MIF) may be the direct target genes of miR-451. MIF could promote tumor growth and invasion [22]. There were over 40% tumor cells decreased with deletion of endogenous MIF in colorectal carcinoma [23]. All these findings indicated that miR-451 may function as a tumor suppressor in various cancers. However, the functions and mechanisms of mir-451 still need further research.

In Conclusion, Down-regulation of miR-451 is associated with the tumor stage and lymph node metastasis in gastric cancer. MiR-451 may be a potential biomarker and potential therapeutic target for diagnoses and prognoses of gastric cancer.

## MATERIALS AND METHODS

### Samples and clinical pathologic data

A total of 420 patients, including 268 patients with gastric cancer and 152 normal patients, who underwent surgery without preoperative treatment at Henan cancer Hospital from May 2009 to December 2014 were included in the present study. The normal patients group was control group. In the gastric cancer group, there were 228 males and 40 females. The median age of the patients was 49 years old (ranging from 35 to 74 years old). The selection criteria for patients were as follows: (1) pathologically confirmed patients with gastric cancer; (2) the patients had no history of other cancers; (3) No patients had preoperative chemotherapy, radiotherapy, or other treatment history. All tissues were snap-frozen in liquid nitrogen and stored at −80°C.

Overall survival was defined as the time from the initially surgery to death of the patient or, in cases of living patients, the date of the last follow-up. The main of follow-up was by telephone. The assisted ways were letters and visits. The follow-up included gender, age, tumor stage, tumor size, lymph node metastasis, survival time, postoperative metastasis and recurrence. The average follow-up time was 48 months (3-60 months). The research protocol was approved by the institutional Ethics Committee of the bioscience of Affiliated Cancer Hospital of Zhengzhou University for medical and health research.

### Quantitative real-time polymerase chain reaction

Total RNA was isolated from frozen gastric cancer tissues by using Trizol agent (Shanghai Sangon) according to the manufacturer's instructions. The expression level of miR-451 was determined by using SYBR® Premix Ex Taq™ II kit (TaKaRa) and U6 small nuclear RNA was used as an endogenous control for data normalization. Relative quantification of the miRNA expression was calculated with the ratio of Ct miR-451 to Ct U6 (as an internal control). All the tissues, including 268 gastric cancer patients and 152 controls, were examined by quantitative real-time polymerase chain reaction. The cut-off value of was 0.01.

### Statistical analysis

All statistical analyses were performed using SPSS 17.0 statistical package for windows (SPSS Inc., Chicago, IL, USA). The Student's t test analysis was performed to compare the clinical characteristics and the expression of miR-451. Survival curve of gastric cancer was estimated by the Kaplan-Meier method. Prognostic factors analyze were estimated by multivariate Cox regression. P≤0.05 was considered to indicate a significant difference.
