# Correction: MiR-451 as a new tumor marker for gastric cancer

**DOI:** 10.18632/oncotarget.27291

**Published:** 2019-10-29

**Authors:** Yong Shen, Jiao-Mei Gong, Li-Li Zhou, Jia-He Sheng

**Affiliations:** ^1^ Department of Pathology and Pathophysiology, Medical School of Southeast University, Nanjing, Jiangsu, China; ^2^ Department of Clinical Laboratory, Affiliated Cancer Hospital of Zhengzhou University, Zhengzhou, Henan, China; ^3^ Department of Clinical Laboratory, The Second Affiliated Hospital of Zhengzhou University, Zhengzhou, Henan, China; ^4^ Department of Hepatobiliary Surgery, Affiliated Cancer Hospital of Zhengzhou University, Zhengzhou, Henan, China

**This article has been corrected:** The authors' affiliation information was listed incorrectly. The proper affiliations are as follows:

^1^Department of Pathology and Pathophysiology, Medical School of Southeast University, Nanjing, Jiangsu, China

^2^Department of Clinical Laboratory, Affiliated Cancer Hospital of Zhengzhou University, Zhengzhou, Henan, China

^3^Department of Clinical Laboratory, The Second Affiliated Hospital of Zhengzhou University, Zhengzhou, Henan, China

^4^Department of Hepatobiliary Surgery, Affiliated Cancer Hospital of Zhengzhou University, Zhengzhou, Henan, China

Original article: Oncotarget. 2017; 8:56542–56545. 56542-56545. https://doi.org/10.18632/oncotarget.17239

